# Tensor language model enables generative scheduling for efficient tensor compilation

**DOI:** 10.1038/s41598-026-41392-8

**Published:** 2026-05-19

**Authors:** Sajid Mehmood, Aqleema Arooj, Ahmad Sami Al-Shamayleh, Samera Batool, Inayat Ur-Rehman, Muhammad Dilshad Sabir, Sabeen Masood, Adnan Akhunzada

**Affiliations:** 1 Department of Computer Science, University of Engineering and Technology, Taxila, 47080 Pakistan; 2 Department of Data Science and Artificial Intelligence, Al-Ahliyya Amman University, Amman, Jordan; 3 Department of Computer Science, COMSATS University Islamabad, Islamabad, Pakistan; 4 Department of Computer Engineering, COMSATS University Islamabad, Islamabad, Pakistan; 5 Department of Software Engineering, Capital University of Science and Technology, Islamabad, Pakistan; 6 College of Computing and Information Technology, University of Doha for Science and Technology, Doha, Qatar

**Keywords:** Engineering, Mathematics and computing

## Abstract

The high rate of increase in the deep learning tasks as well as heterogeneous computing systems necessitates compilers that achieve low compile time and high performance. The current state of the art in the use of tensor compilers is based on exhaustive search, a slow and prohibitive process, or heuristics at the expense of generality and quality of optimisation. The paper presents the Tensor Language Model (TLM), a generative framework of a compiler that redefines the optimisation of tensor programmes as a language modelling problem. TLM is also based on a GPT-2 architecture that is pre-trained on millions of tensor programs coded as compact tensor code capturing operator graphs, hardware metadata, and reconfiguration choices as a sequence of tokens. The model creates optimised schedules of tensors, thus avoiding any search or reinforcement learning at run time. Experimental results using ResNet-50, BERT, GPT-2 and LLAMA-7B indicate that TLM compiles up to 61 times faster than search-based compilers (e.g., Ansor, MetaSchedule) and is up to 2.25 times faster than heuristic models (e.g. Roller), and has similar runtime efficiency. TLM then selects a trade-off seemingly traditional between compilation time and execution performance, a scalable, hardware-agnostic and reproducible generative paradigm of next-generation deep learning compilers.

## Introduction

The rapid explosion in development of the deep learning (DL) resulted in building more complex neural network models that cannot be trained without enormous computational resources^1^. In order to survive the performance requirement of the current deep learning workloads, it becomes essential to produce highly optimised tensor programs that are the low-level code implementations of the base operations like matrix-multiplication, convolutions, and activation functions^2^^3^. To achieve efficient execution, these tensor programs are adapted in specific processor: either CPU, GPU, or dedicated accelerators^4^. Nevertheless, it is a challenge on a large scale to attain optimum performance under varied agendas and hardware capabilities.

Conventionally, deep learning pipelines based on the common frameworks, including TensorFlow, PyTorch, and MXNet, are highly dependent on vendor libraries regarding kernels (cuDNN and oneDNN)^5^. The libraries are offering good performance to common workloads as base libraries offer hand-optimised implementations of tensor operations. Along with its effectiveness, the given approach is also characterised by several limitations. To begin with, the cost of developing and sustaining such libraries is quite expensive in terms of engineering involvement, and a considerable amount of time is involved in most cases, as well as it may involve knowledge of specific hardware^6^. Second, their static kernel libraries are normally specialised to a specific, small set of operators or use cases, which obstructs their generality^7^. There are numerous new workloads or non-standard operators which are not well optimized as there are no suitable kernels.

To remedy these shortcomings, automatic tensor compilers such as TVM, Halide, Ansor, and MetaSchedule have been invented capable of automatically generating optimised tensor programs at high-level computational graphs. The systems rewrite a computational graph to low-level code by searching large spaces of potential code rewrites. The objective is to identify those decisions to achieve an optimal combination of tiling, unrolling, memory layout, parallelization and vectorisation to reduce execution latency^8^. Existing tensor compilers do, however, suffer a deeply rooted efficiency performance tradeoff, not yet thoroughly overcome^9^.

The current solutions can normally be categorised into two groups. Heuristic-driven approaches implement a set of pre-defined constraints to aggressively reduce the search space, e.g. Roller. The technique optimises on compilation time at the expense of possibly missing high-performance implementations thus leading to poor execution performance^10^. Search-based frameworks such as Ansor and MetaSchedule, in their turn, keep a broad search space in order to grab all possible high-performance applicants. Nonetheless, they are based on random sampling together with cost models in assessing and choosing tensor programs. Albeit such exhaustive exploration can yield optimal or near-optimal solutions, compilation time is prohibitively high, sometimes hours and sometimes days on common hardware platforms to obtain a large-scale model like BERT. Such latency places a great bottleneck on deployment pipelines since developers still require a fast and efficient compilation process without affecting the performance of the run time^11^^12^.

Although there is value in the amount of research made, there is one critical gap that has not been addressed; it lies in the absence of generative frameworks for exploring tensor programs^13^. Current approaches are largely based on one of these tactics: random search, properties of heuristics, or evolution, but fail to utilise recent breakthroughs in large language models (LLMs) towards structured generation. Since the results show that language models have performed well in code generation, language structured prediction, and language decision-making processes, there exists a potential unexplored area of inserting the models in tensor program generation. In particular, the semantics of tensor programs can be learned in a language model, serving to inform the decision-making of optimisation, directing it away from exhaustive, but random search with often poor estimates of exploration to drive knowledgeable, context-sensitive inference.

Tensor compilation attempts to convert high-level computational graphs to low-level tensor programs the execution of whose performance is critically sensitive to scheduling choices, such as loop tiling and parallelisation, and memory layout reconfiguration^14^. These choices combine nonlinearly with operator semantics and hardware properties, leading to a combinatorial optimization space, which is too costly to search comprehensively. The current state of the art of tensor compilers is to deal with this difficulty by heuristic pruning or online search based on cost models, resulting in a trade-off between the latency of compilation and the latency of the resulting execution.

This paper restates the use of the tensor programme scheduling as a structured sequence generation problem in this work. The rationale of this formulation is that scheduling choices are natural to formulate as ordered, conditional choices which are operator-structure dependent, and context-dependent on hardware^15^. The compilation process can be autoregressively modelled by expressing the decision and representation of the math program and its scheduling decisions as a concise symbolic sequence of information. Such a view allows the elimination of the iterative search on the internet in favour of direct generative inference at the cost of a substantial reduction in the compilation overhead and with high performance of execution.

In mitigation of this gap, this paper proposes the Tensor Language Model (TLM) Framework, a New tensor program generation system that can turn the problem of tensor program exploration into language generation. The principle behind it is to maintain a large, free decision space that is essential to do well but to take out inefficient sampling in favour of generating decisions based on language models^16^. In that direction we propose a language-model-friendly tensor representation, the tensor language, where the structure of tensor programs (their operator graphs, hardware specifications, and scheduling decisions) is embedded in the form of compact sequences (tensor sentences). Compared to a typical representation of source code, these sentences are much shorter and they are optimised to use token-based models of language.

We obtain a Tensor Language Model (TLM) by using the GPT-2 model to pre-train a million tensor sentences and take on different workloads. The cooperating model is fine-tuned by supervised learning, over demonstration data, which is high-performance tensor programs with measured execution latency. This allows the model not only to discover general optimisation patterns but also hardware specific decision. In contrast to previous approaches we allow offline pre-training knowledge to be used together with online decision-making, leading to a probabilistic but informed generation process that generates efficient tensor programs consistently. Main Contributions

GPT-2 architecture^17^ is selected to be used as the basis of the Tensor Language Model (TLM) because: it is effective in autoregressive language modelling; it shows good performance in structured sequence tasks; and finally, it is easy to implement into the custom training pipelines. Learning complex, multi-level dependencies, as in operator semantics, tensor dimensions, scheduling decisions, and hardware constraints, is a main requirement of tensor program generation, and GPT-2 is capable of modelling these dependencies using self-attention. It is computationally more efficient and capable of being trained on millions of tensor programs with no massive-scale infrastructure than its more recent counterparts (such as GPT-3 or GPT-4^18^) due to its relatively lightweight architecture. Also, as opposed to the most recent proprietary models, GPT-2 is open-source in every aspect and can be customised very precisely to control training, adaptation, and inference procedures. Flexibility is the key to working at the compiler level, where issues of interpretability, reproducibility and hardware integration become decisive. Therefore, GPT-2 has ideal compromises in terms of capacity to model, trainability and applicability to examples of generative tensor compilation.

In this paper, a generative compilation framework is presented and reformulates the problem of scheduling tower programmes as a language modelling problem. The four main inventions of the suggested strategy are listed below. To begin with, this paper formulates the problem of scheduling as a sequence generation problem, where the structure is task-specifically designed to allow autoregressive model types to directly generate scheduling decisions. Second, this paper develops a small and extensible tensor language, in which the organisation of operators, scheduling decisions, and hardware description are combined into a single symbolic expression. Third, this paper uses offline pre-training and then controlled fine-tuning to make the entire optimisation process transition to the training stage, and removes runtime search and cost-model-directed exploration in the compilation stage. Lastly, this paper proves that generative inference is an effective way of eliminating the classical trade-off between compilation time and runtime performance.

Our idea is to have a Tensor-based compiler named TLM, which incorporates large language models to substitute random sampling with an informed decision generation.

- A tensor language is created to encode tensor programs shortly and efficiently, which is suitable for models.
- The suggested method includes a training pipeline that uses offline pre-conceptualisation and then supervised fine-tuning on high-performance tensor programs.
- Compilation is up to 61x faster than Ansor and MetaSchedule, and 2.25X faster than Roller (compiling 1x slower) at the same compile time.
- Extensive testing demonstrates broad scalability for both vision and NLP workloads, in ResNet, BERT, GPT-2 and LLAMA.

This paper presents a compilation framework based on generative tensors that redesigns schedule optimisation as an organised sequence generation, based on several intentional architectural and methodological decisions that allow a compilation to be efficient and scalable. The problem of scheduling is modelled as autoregressive sequence prediction since scheduling decisions inherently create ordered transformation paths where one step is conditioned by the previous choices such that the system will learn implicit optimisation strategies in comparison to explicit search heuristics. The expressions of scheduling actions are represented in a special-purpose tensor language which offers a restricted set of symbols whose grammar ensures the syntactic correctness and is expressive enough to represent intricate optimisation policies. The paradigm of performing optimisation is an offline training paradigm whereby the framework is trained to learn a large set of high-performance schedules that have been gathered previously, and the computational cost of deployment is which is redirected to training, allowing it to generate schedules nearly instantly upon inference. The architecture is also not tied to particular workloads; the trained model can thus learn reusable scheduling knowledge that becomes applicable across operators and models such that a single trained system can be used to support a variety of compilation tasks without specific task retraining.

These design options are a combination that offers a number of technical benefits over current learning-based scheduling methods. The technique removes costly web search, latency in compilation is significantly lowered, and transferable patterns of optimisation have been learned on previous data without incurring the high cost of a competitive runtime behaviour due to schedules generated reflecting statistical regularities in high-quality optimisation paths. The success of the methodology is hinged upon the representativeness of the training corpus and the similarity of deployment workloads with the already observed programmes. In cases where operator patterns and hardware characteristics are sufficiently represented in the training data, the model should generalise well by using learned scheduling structures, but cases where the hardware architecture is significantly different or which involve optimisation primitives not represented by training data should be addressed with further training data.

## Related work

The optimisation of tensor programmes has taken three main directions, including search-based auto-tuning, heuristic and hybrid compiler techniques, and learning-based scheduling models. More recently, generative large language models (LLMs) have been studied as programmable inference engines as part of compilation pipelines. These paradigms are fundamentally different in terms of the modelling assumptions, sources of training signals, inference behaviour and deployment requirements.

Auto-tuners built by search represent scheduling as an organised search through a large combinatorial design space. The follow-ups of previous systems like Ansor and MetaSchedule^19,20^, show that well-designed search spaces and learned cost models can achieve a significant decrease in exploration overhead with a high level of runtime efficiency. HAOTuner built on the prior Work HAOTuner^21^ considers the variability of the tensors by incorporating hardware-aware heuristics in a cost-model-driven search algorithm and demonstrates significant results between runtime performance and tuning latency on a variety of different GPUs. Scheduling is further reinvented by Gensor  ^22^ which is a graph traversal problem that is minimised by Markov modeling allowing the generation of kernels in seconds, and can report 30 percent improvements on ResNet-50 and GPT-2 models. These methods are important for minimising search cost but still rely on operator templates and workload-dependent offline profiling.

Heuristic and hybrid compiler systems focus on the predictable compilation time and deployability. The Hidet project by Hidet^23^ uses a task-mapping paradigm to build scheduling spaces in an organised manner, and its results in order-of-magnitude improvements in tuning time over AutoTVM and Ansor. Equally, oneDNN Graph Compiler  ^24^ combines both primitives that are designed by experts and graph-level optimization such as fusion, layout transformation, and quantisation. Another example of industrial systems integrating hand-written kernels with hardware knowledge is industrial systems like TensorRT and CUTLASS  ^25,26^ to provide state of the art inference throughput. Though very practical in use, such systems are based on managed knowledge of experts and are often limited to where one can see or where the hardware is in a homogeneous environment.

The scheduling methods that are based on learning introduce a new abstraction in that the process of scheduling is modelled as a decision-making process based on data. Sequential policy learning Schedulers based on reinforcement learning (RL)-based assigns optimisation as sequential policy learning, in which a neural policy chooses scheduling actions and gets reward signals as a result of cost models or empirical runtime measurements. These methods have the ability to find quality schedules, but they usually need to be trained with iterations and many interactions with compilers or simulators. Imitation-learning methods rather make use of expert schedules or successful search paths as supervision indicators, which lowers the cost of exploration at the expense of still relying on handcrafted representations and data-specific workloads. Inference, in both instances, usually has the form of step-wise decision generation based on intermediate states.

More recently, huge language models have also been added to the compilation workflow. Compilation aided  ^27^ Shows that GPT-4 can compile code at the accelerator level by prompting with structured pipelines, which can cut manual engineering work. AutoComp  ^28^ uses LLMs to optimise GEMM and convolutions, achieving huge speedups compared to vendor baselines. These works also demonstrate the generative ability of LLMs but are mostly concerned with instruction-level code generation and not systemic scheduling of tensors and frequently need prompt engineering or structural constraints to be accurate.

Compared to the explicit sequence-based scheduling models of RL-based or imitation-learning schedulers, the suggested Tensor Language Model (TLM) can redefine the scheduling process as autoregressive sequence generation of symbols over a unitary representation of operator structure, hardware context, and scheduling choices. Training is done fully offline using high-performance scheduling information and inference generates a full schedule in one forward pass without any runtime exploration, reward evaluation, or rollout. This change in models moves the complexity of optimisation to pretraining, which can be deployed deterministically and with low latency with all the competitive performance of online search.

The conceptual differences between the modelled paradigms are outlined in Table  1 of the comparative analysis of several representative paradigms in terms of modelling formulation, training signal provenance, inference mechanism, and deployment characteristics.

**Table 1:** Conceptual Comparison of Tensor Program Optimisation Paradigms.

Approach	Modeling Formulation	Training Signal Source	Inference Procedure	Deployment Characteristics
Search-Based Auto-Tuning (e.g., HAOTuner, Gensor)	Structured search over schedule space with cost modeling	Offline profiling and performance measurements	Iterative exploration during compilation	High runtime efficiency; per-workload tuning required
Heuristic / Hybrid Compilers (e.g., Hidet, oneDNN, TensorRT)	Rule-based or expert-designed scheduling templates	Expert knowledge and handcrafted primitives	Deterministic rule application	Low compilation latency; limited flexibility to unseen operators
RL-Based Scheduling	Sequential policy optimization over scheduling actions	Reward signals from cost models or runtime feedback	Multi-step policy rollout	Requires exploration during training; interaction with simulator or cost model
Imitation-Learning Scheduling	Supervised action prediction from expert trajectories	Expert schedules or search-derived datasets	Step-wise action decoding	Reduced exploration cost; generalization depends on dataset coverage
TLM (Proposed)	Autoregressive symbolic sequence generation	Offline high-performance scheduling corpus	Single-pass schedule generation	No runtime exploration; deterministic low-latency inference

Overall, the current state of tensor scheduling frameworks mirrors one of the key trade-offs between the quality of search, the time of compilation, the flexibility of models, and the complexity of deployment. Search-based searching approaches are fast in runtime, with overhead of exploration, in contrast to heuristic searching algorithms, which are predictable in their latency, but not as adaptable as learning-based searching algorithms, which are data-driven but may require sequential decision modelling or even feedback during execution. Generative LLM-based methods have shown encouraging reasoning performance, but largely they are oriented towards code synthesis, as opposed to a form of structured scheduling. The suggested TLM is placed in a complementary role in this landscape in that the operator structure, hardware context, and scheduling decisions are integrated together within an autoregressive symbolic model. The idea of moving optimisation burden to large-scale offline pretraining and allowing single-pass schedule generation during inference time is meant to make the approach both efficient in deployment, as is done by heuristic, and performance-competitive, as is done by search-guided ones, without introducing runtime exploration dependencies.

## Background and motivation

### Tensor compilers

The Tensor compilers are very important in mediating the divide that exists between the high-level deep learning frameworks and the low-level hardware execution. In contrast to the use of general-purpose compilers that is required in traditional programming models, tensor compilers are optimised to work on tensor computation, which is the fundamental construct in the neural network. Automatic tensor compiler systems, such as TVM^29^, Halide^30^, or AKG^31^, generate optimised code, running on GPU or CPU hardware, or AI accelerators, to implement tensor operators.

Compilation Generally, compilation entails manipulating computational graphs into hardware-appropriate tensor programs through successive optimisations. These are loop tiling, unrolling, vectorisation, memory layout, and parallelisation strategies. It aims to finish the actions as fast as possible, capitalising on the usage of hardware and striking a rewarding balance between processing power and the memory bandwidth. But the combinatorial and large space of possible optimisations renders this a truly computation-intensive task. Restrictions of the existing tensor compilers overcome this only through hand-tuning heuristics, which may produce suboptimal results, or search-based approaches, which are costly to compile using many samples.

### Role of tensor programs in hardware acceleration

The most fundamental part of a deep learning model, from an execution perspective, comes in the form of tensor programs, which are the low-level application of functions, such as matrix transportation, convolution, pooling as well as activations. The Tensor programming features the mapping of operations onto hardware resources such as threads, cores, vector units, as well as memory hierarchies. A key requirement on the road to hardware acceleration is efficient tensor programs, important in particular when targeting heterogeneous platforms (like GPUs, CPUs, TPUs, and custom AI accelerators).

Hardware acceleration Hardware acceleration necessitates the use of device-specific features by the tensor programs; this includes SIMD vector instructions, GPU thread hierarchies, or custom compute units like Tensor Cores by NVIDIA. Consequently, the tensor program generally influences the runtime performance of the deep learning treatments and thus has a direct effect on the quality of the same. A badly mongraphicite optimized tensor program may fail to take full advantage of the available hardware, may suffer cache misses, and may impose unnecessary synchronisation overheads; performance can be dramatically hurt.

### Language models and learning-based optimisation

This is because language models (LM), especially large-scale models such as GPT-2, GPT-3 and ChatGPT, have introduced broad enhancements to the natural language processing domain due to the capability to create reasonable and contextually-informed text. They are usually trained through Causal Language Modeling (CLM), which is to predict the next token of a sequence based on its prior tokens. Lots of datasets and parameters, billions in fact, are used to train the models, and this enables the models to acquire structural and semantic information about language.

The alignment of the language models to the desired outputs has been even enhanced recently by instruction tuning and reinforcement learning with human feedback (RLHF). Through the methods of Supervised Fine-Tuning (SFT), RLHF techniques that are adapted in systems like InstructGPT and ChatGPToptimised enable result specification to the given task or domain.

Although one normally uses LLMs to work with natural language, the fact that these models can deal with structured sequences and problems of decision-making suggests that they can be applied to code generation and optimisation in a promising manner. Applications to the problem of generating programs written in tensor languages, though, are specific to language modelling in that they must deal with long sequences, must conform to strict syntactic specifications, and must include hardware-specific knowledge. To resolve such drawbacks, the work is founded on a language embedded in a special language model. Applying our work, a language model generates scheduling decisions and efficient optimisation. Modelling of tensor programs is turned into a structured sequence generation problem.

### Terminology and conceptual scope

In this section, the terminology in the paper is explained to prevent ambiguous terms, as well as to be quite specific to define the technical scope of the essential notions. To represent multi-dimensional data objects in abstract algebraic terms, the term tensor is used in this piece of work to refer to structured multi-dimensional data objects when presented in compiler intermediate representations, and to operator descriptions, as opposed to abstract mathematical tensors presented in abstract algebraic notation. It is centred on tensors, which are programme-level objects, whose shapes, memory layouts and access patterns are what dictate how they will be optimised in a compilation process.

A tensor program refers to a compiler-level description of one of a series of tensor computations, including both its operators and loop structures, its memory accesses and its transformation primitives. Such programs specify computation semantics and optimization opportunities, which act as the input abstraction of scheduling.

The formalised symbolic representation of scheduling the actions and sequence of transformation is known as the tensor language. This language characterizes a structured token space whereby every token represents the valid compiler transformation/scheduling directive. Its grammar only allows generation of syntactically and semantically valid optimization sequences.

The generative model which is trained on sequences in this tensor language is called the Tensor Language Model. It acquires statistical knowledge of high-performance schedules and generates entire scheduling programmes in an autoregressive fashion. The model instead uses compiler representations instead of raw numerical tensor data.

These definitions define that the terminology is consistently applied to a system and compiler setting. Whenever the term tensor is used in the later sections, it is taken to mean compiler-level compile-time tensor programme constructs unless otherwise indicated.

## System design and methodology

### Overall architecture

**Fig. 1:**
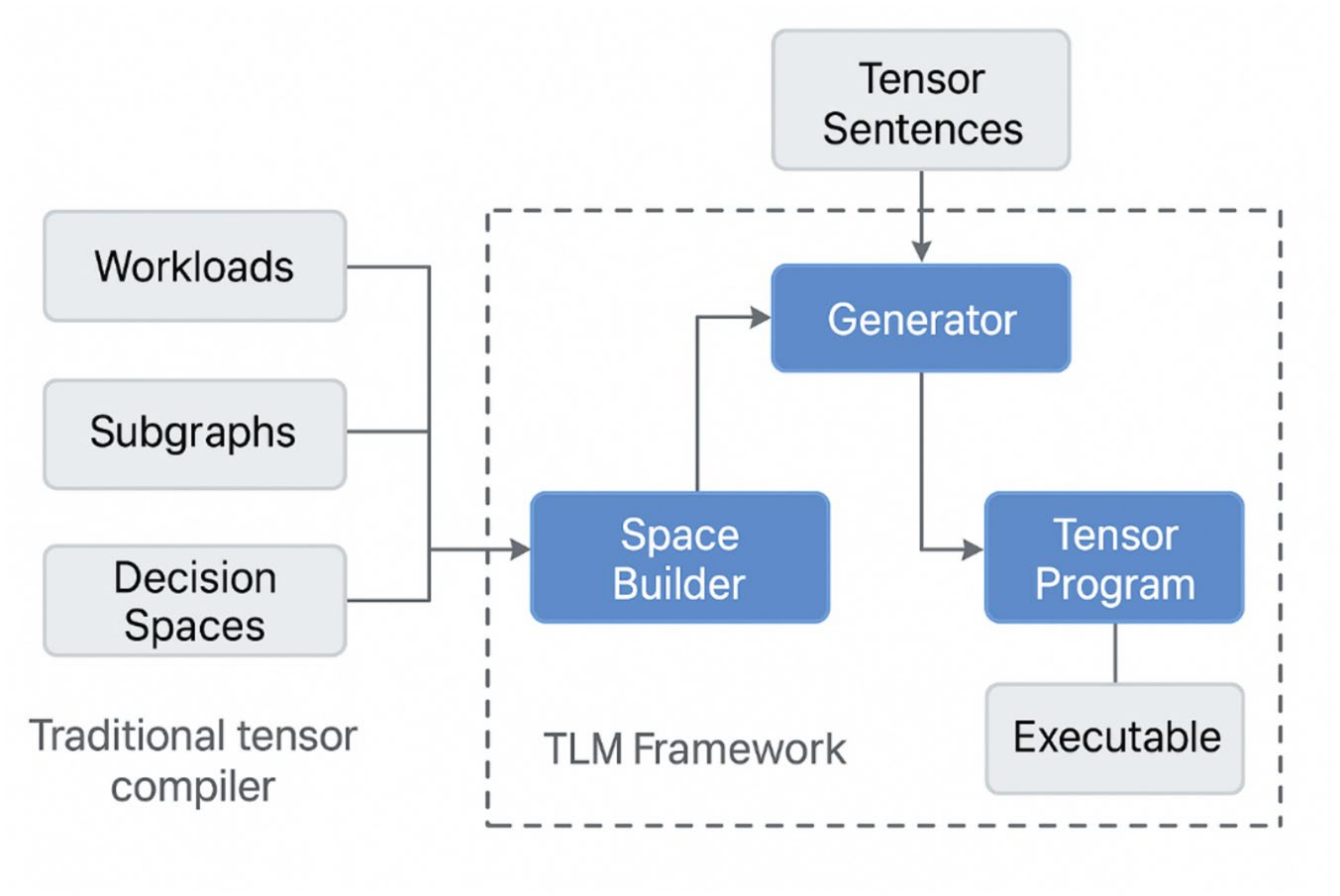
TLM framework overview: Space Builder constructs decision spaces; Generator creates optimised tensor programs for execution.

The main component in the Tensor Language Model (TLM) framework Fig. 1 is developed to replace the generation of the tensor programs that usually relies on methods of a classical search problem but is rather to become a language model-based generation. Said framework is composed of two major components namely the Space Builder and the Generator. These elements collaborate with each other yet exist independently and this enables the system to have a vast exploration space and high efficiency in the generation of programs as well.

The Space Builder is in charge of the definition of the search space of tensor programs. This element accepts as input the computational workloads in some form, which most commonly is a series of computational graphs and breaks these down into computationally independent subgraphs that may then be separately optimised. To each subgraph, the space builder creates a detailed decision space listing all configuration possibilities of the generation of tensor programs. Among these decisions there are tiling factors on loop axes, unrolling strategies, memory layout conversions, thread and block bindings to be executed on the GPU and vectorisation instructions. The large exploration space explicitly retained in the system assures that it does not prune early the possibility of high-performance implementations by performing heuristic pruning. This design decision maintains the flexibility required in order to have state-of-the-art performance with a wide variety of workloads and hardware platforms.

After the decision space is defined by the space builder, the work of program generation is handed off to the Generator, which is driven by the Tensor Language Model (TLM). Rather than carry out random sampling or multi-pass iterative searches such as the sample-rich donor search, the generator produces one or more tensor sentences using a pre-trained and fine-tuned language model which has been trained on tensor programs. In autoregressive decoding, the generator predicts each generated decision in the tensors sentence consecutively on the basis of the tensorinput sub-graph, hardware specifications, and previously generated tokens. This is iterated until a full tensor program has been create.

With the decoupling of the roles of space builder and generator, the TLM framework provides a decoupled architecture that reconciles the exploration capacity and efficient decision making. The space builder ensures that the system keeps access to all optimal configurations of high performance and the generator is limited to generating optimised schedules based on what has been learnt from previous data. The architecture enables the TLM framework to generate both fast-performance tensor programs and a low compilation time as compared to the conventional search-based compilers.

### Terminology and conceptual clarification

This work uses several words with certain technical definitions associated with tensors and tensor programmes. We have explained the terms used within the paper to prevent any ambiguity.

To begin with, mathematical tensors define a multidimensional array and its algebraic operations in terms of linear algebra and numerical computing. Although such tensors are the data on which machine learning models operate, this is not what the models model in this study.

Secondly, a tensor program is a low-level or operator-level program that describes the execution of a computation on hardware on a tensor. A tensor program encompasses both the mathematical definition of an operator (e.g. convolution or matrix multiplication) and a collection of scheduling choices, including loop transformations, parallelisation choices, and memory access patterns, all of which are known to dictate performance.

Third, a linearised tensor and symbolic form of tensors programmes and their schedule description is called the tensor language. The structure of operators, shapes of tensors and scheduling decisions, as well as other hardware-related metadata are encoded in this representation as a sequence of discrete tokens, allowing sequence-based learning and generation techniques to be used.

Lastly, there is the Tensor Language Model (TLM), which is an autoregressive generative model that is trained on the tensor language representation. With an operator specification and hardware context, the TLM produces a series of scheduling decisions, which as a unified definition create a tensor program. Throughout this paper, the term tensor otherwise indicates operator-level tensor programmes and their scheduling descriptions, as opposed to the use of the term in a theoretical context to describe mathematical objects with tensors on their values or to describe mathematical modelling that uses tensors.

The general idea behind the proposed framework is that high-performance tensor schedules have repetitive structural patterns that can be learnt using offline data. The approach explores global coherent schedules by modelling scheduling as sequence generation instead of policy optimisation or search which exploits these regularities to generate world-aware schedules in a single inference pass. Such design selection distinguishes the proposed approach from the existing learning-based scheduling frameworks which are based on iterative decision-making, explicit modelling of rewards, or even per-workload policy adjustment.

### Mechanistic analysis and applicability discussion

The practicality of the given generative scheduling formulation is due to its capacity to represent compiler optimization as a structured sequence prediction issue with scheduling decisions that vary in strong statistical regularities among operators, architectures, and effort. Learning in the search space is performed onlineoptimisation but the model internalises recurring patterns of optimisation during offline training and applies them at inference time. This moves the computational cost of exploratory runtime to a single-time training cost, which allows much faster schedule generation without sacrificing the quality of performance.

In modelling terms, schedules in tensor programs have a composition structure in which transformations like tiling, fusion, reordering and parallelisation are guided by restricted syntactic and semantic rules. The abstract model of schedules as sequences of tokens enables the model to decompose local dependencies between closely related scheduling choices and long-range dependencies between the optimisation trajectory as a whole. The generative formulation thus acquires tacit heuristics that need to be rediscovered time and time again by the traditional search-based systems.

The method is most effective when the training corpus represents the variety of operator patterns, hardware features and optimisation schemes adequately met during deployment. In these circumstances, the model generalises through the projection of unknown programmes to previously acquired scheduling patterns. Performance varies based on the resemblance between new programmes and structures that have occurred before when the difference between the deployment and training distribution is significant. The underlying assumption of the approach in this behaviour is that high-performance schedules exhibit transferable structural patterns across workloads.

The framework is based on a number of assumptions. First, the scheduling language provides a valid and expressive action space the syntax of which restricts generation to semantically correct programmes. Second, the offline data gives good supervision indications that are indicative of optimal schedules. Third, the hardware target is contained in the distribution of architectures executed in training. Ifoptimisation these assumptions are satisfied, the generative approach offers an efficient substitute to search-based optimization because it amortises the cost of scheduling over a large number of instances of compilation.

This discussion explains that the advantage of the model is not only brought about by faster inference, but it is also brought about by learning of reusable optimisation knowledge, which learns latent regularities in the compiler design space. Based on this, the approach complements and does not substitute traditional search-based methods, and is especially beneficial in applications of repetitive compilation of dissimilar workloads on fixed hardware targets.

### Generative modelling perspective on tensor scheduling

The rationale behind the suggested approach is that the decision-making process of scheduling tensors has very strong statistical regularities between the workloads and hardware platforms. Similarly shaped and patterned computational operators can be well matched by similar schedules, and patterns are replicated across models and domains. These regularities are hard to write down using heuristics, and expensive to find using a search engine, yet they can be learnt efficiently using data.

Autoregressive language models are very appropriate in the capture of these regularities since they learn conditional dependencies in the order of elements. In the application to the scheduling of tensors, every scheduling decision is produced under the influence of already generated decisions, operator metadata, and hardware specifications. This enables the model to learn interrelations implicitly among a variety of optimisation dimensions - tiling, parallelism, and patterns of memory access - that are hard to optimise singly. Consequently, it is possible to perceive the problem of generating a globally coherent schedule as conditional generation, with the goal of only a single inference pass to generate a globally coherent schedule.

### Space builder

Large exploration space denoted S is at the centre of tensor program generation. It is the set of every valid possible implementation of a particular tensor operation taking into account all optimisations that can be made to a given hardware. More formally the exploration space S may be described as a set of sequences of scheduling decisions Eq (1):

1 $$\begin{aligned} S = (s_1, s_2, \ldots , s_n) \end{aligned}$$

in which the $sis_i$ is a sequence of choices that specify how to plan the computation. All sequences contain tiling factor, loop unrolling, parallelisation, and memory layout transformation decisions. All these decisions impact the way that tensor program interacts with specific hardware components including caches and SIMD, threads, and shared memory.

Example: Consider the case of a nested loop that performs a matrix multiplication. Here the space builder will have to evaluate all the possibilities about tile nesting loops, axes swapping, computation to thread or core assignment and memory accesses alignment to coalesce loads. Axes of loop. The combination of the dimensions of a loop and the hardware that the loops will use grows exponentially and is therefore not practical to exhaustively enumerate.

**Fig. 2:**
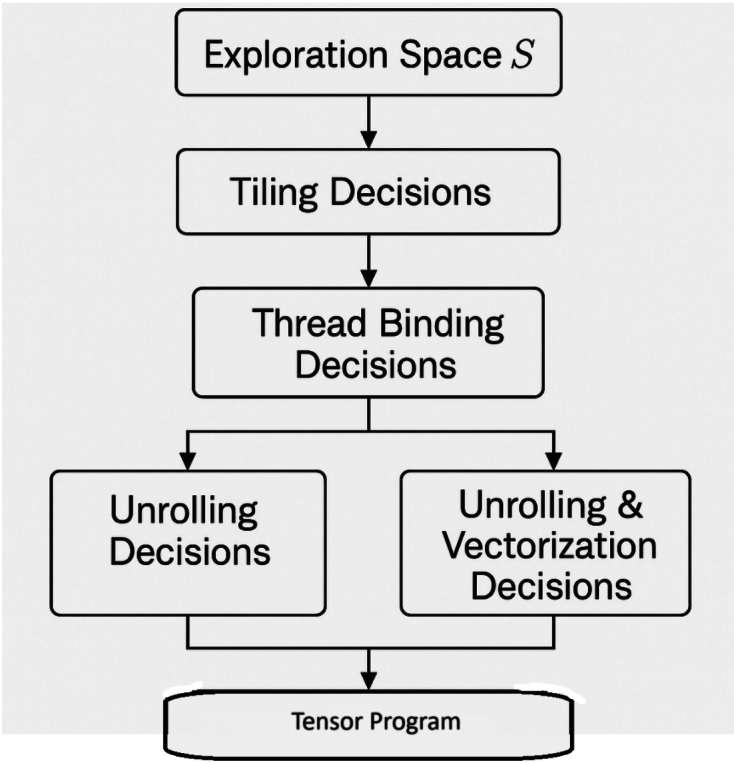
Space Builder pipeline: Constructs exploration space with tiling, thread binding, unrolling, and vectorization decisions for tensor program generation.

In the TLM concept, the Space Builder pipeline Fig. 2 separates the exploration space from decision-making. It applies domain knowledge and operator semantics and generates the search space, and does not preselect high-performance options early in the search. In particular, the Space Builder produces: Tiling factors: The splitting of loop axes into minor units aiming at better usage of cache and memory bandwidth.

- Thread binding alternatives: The iteration of mapping the loops to GPU threads, GPU thread blocks or CPU cores.
- Unrolling decisions: The decision as to which loops to unroll to minimise control overhead, and to reveal instruction-level parallelism.
- Vectorization flags: Allowance of whether to vectorise the loops that are to perform SIMD execution.
- Memory access patterns: Data layouts are being reordered in such a way that data placement corresponds with the requirements of hardware memory hierarchies.

Building this decision space in full in advance, the framework retains the expressiveness and performance upper bound of search-based approaches, but allows the Generator to make efficient selection out of this space.

The Space Builder is tasked with the responsibility of building the organised time space of a particular tensor programme. There is a set of valid scheduling dimensions and constraints, which are determined by the Space Builder over specified operator specifications, shapes of a single parameter in the tensors, and the target hardware metadata. They are loop transformations (e.g., tiling and unrolling), parallelisation approaches, possible ways of vectorising, use of memory hierarchy, and transformation of data layout. Instead of listing the complete combinatorial space, the Space Builder can specify an ordered decision space which represents possible scheduling decisions without violating semantic and hardware constraints.

Symbolic representation is used to represent each scheduling decision, which is arranged as a deterministic sequence of decision slots. This formal representation has two purposes: the representation imposes validity on schedules generated, and it offers a homogeneous interface between the compiler front-end and the generative model. The result of the Space Builder is a partially specified programme of tensors and a serial form of the scheduling decision space.

### Generator

Generators are an inference system that can be described as the autoregressive model that is based on the Tensor Language Model. The Generator uses the operator-level and the hardware metadata in addition to the serialised representation generated by the Space Builder to generate scheduling decisions on a token-by-token basis. The model makes a scheduling decision at each phase based on the decisions previously arrived at, making decisions coherent across optimisation dimensions that cannot be independently optimised.

The Generator produces a full sequence of scheduling tokens, called a tensor sentence, that completely specifies a legal schedule of the input tensor programme. This code ismodels in turn compiled and converted by the compiler back-end into an actual implementation at low-level code. The Generator creates schedules by one-way pass, thus removing iterative search, runtime cost analysis or refinement of schedules at the time of compilation.

### Interaction between space builder and generator

Space Builder and Generator work closely but loosely. The Space Builder determines the structural constraints and decision order of the scheduling problem and the Generator trains to instantiate concrete scheduling decisions within this structure using offline training. Such a separation enables the system to be correct and flexible: any changes to the rules of scheduling or hardware targets can be reflected by updating the Space Builder without re-training the generative model.

### Tensor language design

**Fig. 3:**
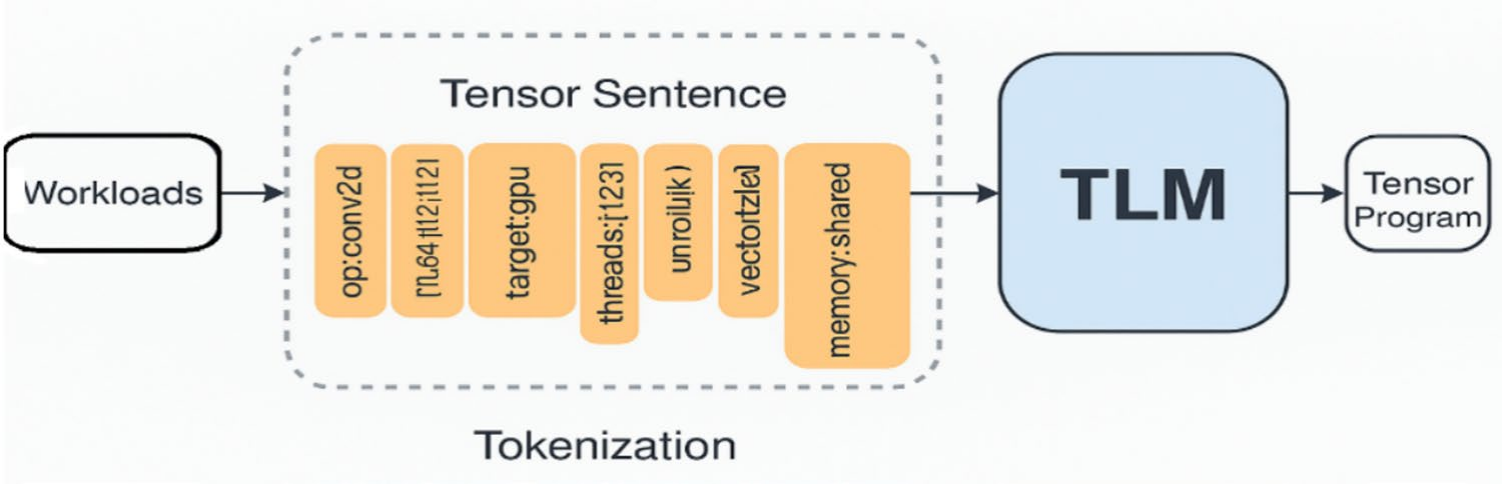
Tensor Language Model pipeline: Workloads are tokenised and human-readable into tensor sentences, processed by the TLM, and transformed into tensor programs.

To ensure that language models like transformer can consume the exploration space, the TLM framework presents a Tensor Language Fig. 3, which is effective in that it represents an orderly sequence in a language-like model and functions through token-based generation. Traditional representations of tensor programs e.g. in the form of source code, intermediate representations (IR), do not very well support language models because they are long, they are often highly complex, and they are heavily hierarchical. Thus TLM describes a flattened human-readable tensor-sentence that contains all the information of interest towards generating a program.

A tensor sentence is a series of tokens, which stands in a map-to relation to:

- Operator Graph Metadata: Operator type (for example, conv2d, matmul), tensor dimension, and shape information.
- Hardware Context: Single out target specifications of the hardware e.g. number of cores, size of cache, GPU thread hierarchy.
- Scheduling Decisions: The tiling size, loop orderings and unroll factor, thread, and memory layout decisions.

e.g. imagine a simplified human readable version of a 2D convolution sentence:
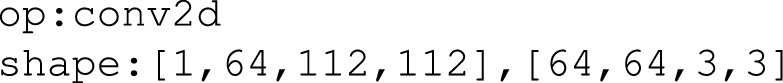

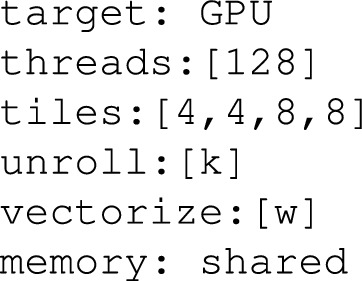


This is a sentence defining a convolution of particular tensor shapes (they have to be targeted at being run on a GPU with 128 threads). Each of the scheduling decisions consists of tiling by loop around the spatial dimension, unrolling along the kernel dimension, vectorisation along the width dimension and shared memory use.

By providing a representation of tensor programs in this compact, sequence-friendly form, the tensor language not only fills the gap between compiler IRs and language models but also allows efficient learning and generation using sequence modelling techniques. The design specification has it that the whole decision process can be contained in a normal length sequence limit of 1024 tokens, which means it is adaptable with the standard autoregressive language models.

To judge this concept more fully, consider the following example: Conv2d action on a 4D tensor on a GPU using a specified thread hierarchy. Such an human-readableoperation may be expressed in a conventional IR as a node in a computation graph connected to input and output buffers with shape and datatype metadata. In TLM, though, we tell the same information in something resembling a natural-language sequence and obey a structured grammar. For example:
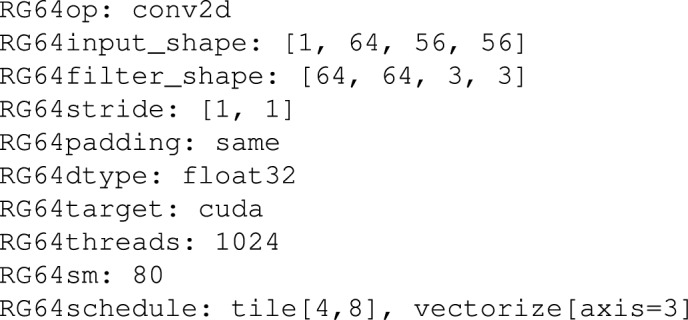


Besides encoding the operator (conv2d) and its parameters (input and filter shapes, stride, padding, data type), the tensor sentence also encodes the hardware-specific context (cuda, the number of threads, the streaming multiprocessors) and the scheduling decisions used (the tile, the vectorise, the unroll and the bind). The fields are all tokenised in a uniform order and delimited by semantic delimiters (e.g., |) and arranged in a logical way to maintain a logical connection between logical operations and their eventual physical deployment.

The design philosophy in this case is to build a semi-natural, compiler-readable language that could be used to make the LLM learn some complex correspondences between the structure of operators and effective scheduling procedures over particular backend hardware. Encoding both syntactic and semantic information in a linear fashion converts the task into a sequence modelling one, by having each token made by a model that predicts the next decision in the pipeline. This allows modellingto cast tensor compilation as a type of conditional generation process, informed by both workload characteristics and characteristics of the hardware.

In addition to that, the tensor language is extendable. New operators like, among others, $batch_matmul$, $layer_norm$, $depthwise_conv2d$ can be included merely by appendingus operator tokens and corresponding parameter schema. Similarly the vocabulary of target and hardware tokens can be extended to support new backends (e.g., Vulkan, ROCm). The modularity of the language in question means that operator semantics as well as optimisation behaviour across domains are naturally captured in token embeddings.

Such tensor sentences are derived in the millions during model training, assembled across and across a wide variety of workloads and devices. Such sequences are subsequently utilisedvisualise to educate the TLM through common autoregressive objectives, and configurations in turn enable the same to forecast superior-quality enabling of schedules on new tensor sentences at the inference stage. This language model is, in fact, learning syntax: it is learning the statistical regularities of what is good scheduling including how specific operators respond to specific patterns of tiling or unrolling them, given different thread constraints.

In order to facilitate interpretability and debugging we also give tools that visualize the decoding of TLM, so that a user can see how a schedule is being made, token by token. As an example, it is possible to look at why the model selected tile[4,8] as input shape and verified that with our understanding of optimal shapes to coalesce memory or reuse shared memory.

To conclude, the success of TLM is based on the use of the tensor language. It provides scalable, interpretable and learnable embedding between the high-dimensional space of operator graphs and the word-based framework of language models. It helps to give a rich yet tractable representation that enables the model to generalise across operators, devices and workloads, and comes up with efficient and deterministic schedules.

### Hardware awareness and generalisation

The suggested framework decouples hardware awareness and hardware specialisation. The symbolic metadata that is added to the tensor language representation consists of hardware traits to the full extent that the same model architecture and generation process can be applied to different targets. Such adesign allows keeping the compiler hardware-neutral at any given architectural level but conditioning the scheduling decision-making on hardware context.

Concurrently, the schedules obtained are as good as the representativeness of the training data. Although the model will be able to generalise search-based to workloads not directly observed in training, no performance on previously unobserved hardware architecture can be assured with further fine-tuning or exposure to such hardware properties. It is a limitation, but it is due to the data-driven nature of the approach and not the reliance on hard-coded hardware rules.

### Tensor language model (TLM)

The Tensor Language Model (TLM) was used as the Generator of the framework, and it generates valid and effective tensor sentences to produce optimised tensor programs. The TLM is initialised with the architecture of GPT-2 that has the small version (12 transformer layers, 768 hidden units, and 12 attention heads). Such a model size is not too expressive but allows reasonable training time and can be implemented on a relatively small hardware without compromising the generation quality.

#### Offline learning and inference-time schedule generation

Tensor Language Model builds on the concept of offline learning to pass the optimisation cost over a significant set of optimised tensor programmes. In pre-training, the model is trained on general structural relationships between operator characteristics, hardware context and scheduling decisions. Further fine-tuning under supervision steers the model to produce schedules that are linked to low execution latency.

The proposed framework solves the challenges of optimisation by devoting the entire effort of the optimisation to the offline stage, avoiding runtime exploration, training the cost models or refining the schedules iteratively. The compiler produces a full sequence of scheduling decisions in one forward pass, which allows it to compile very fast and deterministically, and still maintains performance on par with search based approaches. modelling

#### Distinction from policy-based and imitation-based scheduling frameworks

Even though the proposed training pipeline takes advantage of offline pools of high-performance examples of scheduling, the framework is conceptually different to the existing policy-network-based and imitation-learning-based scheduling models. This paradigm model. Traditional methods usually represent scheduling as a succession of environments-interacting actions, typically the need of explicit state representations, explicit reward signals, or the need to optimise the compiled code in an iterativeenvironment-interacting manner.

In contrast, the Tensor Language Model is only operative in the space of symbolic sequence modelling. The decision to schedule is produced in an autoregressive way as a structured sequence with the option based on operator specifications and hardware metadata. This architecture eliminates the use of online feedback, simulation of the environment as well as policy adaptation at instances. The model directly generates a globally-inconsistent schedule, which is available at inference time, with deterministic and low-latency compilation, and with the same performance as learned on offline data.

#### Pre-training

The procedure of pre-training initialises the TLM on a big set of corpora of tensor sentences gathered over diverse workloads and designs. The training data here covers both suboptimal and optimal tensor programs and it allows the model to learn general structural patterns in the process of generating tensor programs. The pre-training is supplied through the causal language modeling (CLM) task, in which the model is trained to predict the following token, given a flow of preceding tokens.

With the pre-training process, TLM creates a basic grasp of the tensor operations, scheduling judgments, and configurations of quantities. It encapsulates typical patterns within tensor programs, and constructs an internal model of the decision parameters that impact the performance of these programs without ever having seen performance-labelled data.

#### Supervised fine-tuning (SFT)

The model is then Supervised Fine-Tuned (SFT) on a hand-picked collection of good-performing tensor programs. These are gathered by past optimisations and benchmarks, in which every sentence of tensors is tagged with the measures of the execution latencies. Fine-tuning trains the model that will produce a decision sequence that maps to low-latency executions and moves it on to performance-aware generation.

The TLM eventually will not require any runtime measurements to train the cost model because its train takes place offline as opposed to the online cost model training required by traditional search-based compilers.

#### Avoiding reinforcement learning

One example where we do not use RL, despite some successes in large language models applied to dialogue alignment (e.g. RLHF ), is tensor program generation. This needs RL to spend a lot of time communicating with the environment, gathering and running algorithms to receive latency rewards, whereas it is not affordable foris tensor compilers. Also, RL offers instability and only careful engineering of the reward can be operated on which can not be easily addressedvast-scale in hardware specific optimization area. The TLM requires no vastly scale feedback loops at run-time and delivers high performance through purely offline pre-training and supervised fine-tuning.

## Implementation details

Even though the Tensor Language Model (TLM) framework is conceptual, it is still necessary to outline how the approach would be used in practice to compile the dataset, perform training, specify the hardware, and the run-time environment. Our implementation is reproducible, scaleable and general, over a wide variety of workloads and hardware backends.

### Tensor language model architecture

The Tensor Language Model (TLM) is implemented with the GPT-2 small model in the present implementation. In particular, the model is an autoregressive transformer with multiple layers with standard self-attention blocks, whose parameters are fixed in terms of the number of layers, attention heads, and hidden dimensions. The model is not only trained with a causal language modelling objective on top of the tensor language representation, but also predicts scheduling tokens contingent on operator specifications and hardware context.

GPT-2 is chosen as the backbone architecture as it can be successfully used to model autoregressive sequences, and it is open-source, which makes it easier to reproduce the results. Notably, the specified framework is not necessarily associated with GPT- 2. The formulation of the tensor language and training pipeline modelling model-agnostic and can, in principle, be implemented in any other autoregressive transformer architecture with no changes to the system design.

### Formation of datasets

In need to train the Tensor Language Model, we are building a broad dataset Table 2 based on Model Zoo workloads in both Computer Vision (CV) and Natural Language Processing (NLP). Its data set contains common benchmark models, including ResNet-50, MobileNetV2, and EfficientNet vision models and BERT-base, GPT-2, and LLAMA-7B subgraphs that complete language modelling tasks. These models are selected so that there is a diversity in the operator patterns, the dimension of the tensors and the computational nature of the model.

Every model is used to break up into subgraphs and each subgraph represents a collection of tensor operations that may be optimised as a unit. To every subgraph, we come up with several tensor sentences that represent differences in scheduling choices, hardware objectives, and execution contexts. The data comprises about 3.2 million tensor sentences, which were gathered during previous search-based optimisations and due to hand-written expert annotations. This both covers standard and edge-case optimisation cases.

**Table 2:** Dataset Summary for Tensor Language Model (TLM) Training.

Category and Workloads	Subgraphs / Tensor Sentences	Purpose and Models
Computer Vision (CV)	1,500 subgraphs / 1.2M tensor sentences	Pre-training and fine-tuning
*Workload: Image classification*		ResNet-50, MobileNetV2, EfficientNet
Natural Language Processing (NLP)	1,800 subgraphs / 2.0M tensor sentences	Pre-training and fine-tuning
*Workload: Language modeling*		BERT-base, GPT-2, LLAMA-7B
Total	**3,300 subgraphs / 3.2M tensor sentences**	**Model training across six models**

Significant values are in [bold].

In order to label the fine-tuning dataset, we compare every single tensor program with real hardware and obtain the related latency values. The most successful schedules are kept as positive demonstrations and allow the model to have real-life examples of how to have a high-performance decision sequence. This subset of examples constitutes the supervised fine-tuning (SFT) set that will be utilised in orienting and modellingoptimised the model to its performance objectives.

### Model training

We would like to label the fine-tuning set by comparing each of the tensor programs with real hardware and we record the associated values of latency. We keep the most successful schedules in the form of positive examples and give this model actual examples of the successful decision sequence pattern. This set of demonstrations constructs the supervised fine-tuning (SFT) set, which helps in the alignment of the model. The Tensor Language Model is an implementation of the GPT-2 small architecture, which has 12 transformer layers, a 768-dimensional hidden state, and 12 heads of attention. The configuration has been chosen on the balance between the number of models and computational performance that would enable the framework to scale to large datasets whilst being actually applicable to implementation.

The TLM is additionally optimised with the aid of supervised learning on offline sets of high-performance tensor schedules during training. GPT-2 common training setups are also embraced such as causal attention masking and cross-entropy loss on the prediction of succeeding- tokens. Network hyperparameters such as the number of transformer layers, attention heads, hidden dimensions as well as optimisation settings are detailed to facilitate reproducibility.

The pre- training is done with the help of a conventional causal language modelling (CLM) goal. Modelling happens with the domain of 3.2 million tensor sentences, where it is taught to forecast a subsequent token in the sentence of tensors based on the preceding sequence. The process enables the model to approximate structural regularities in the generation of tensor programs in terms of operator ordering, hardware-aware scheduling choices and relation of parameters. The training is done on one NVIDIA V100 GPU (32 GB memory) in 10 hours of pre-training. Our AdamW optimiser has a learning rate of 5e-5 and the batch size of 128, and a maximum sequence length of 1024 tokens.

Upon pre-training, the model is finetuned on the labelled high-performance tensor sentences. The process of fine-tuning involves the adoption of Supervised Fine-Tuning (SFT) which takes place to match the model generation with efficiency in executing the schedules with the best performance involving the scheduling of the datasets during the construction of the dataset. The fine-tuning has quite a good speed when compared to the pre-training, which needs 10 minutes of training in the same hardware conditions. Fine-tuning the dataset is relatively small, highly structured and as a result, the training can be stabilised faster and the behaviour of the generation is predictable.

In the training pipeline we do not use reinforcement learning (RL) because this can easily result in the high costs of training online and the compilation loops that are associated with RL-based reward signals. Rather, training is done entirely offline, where the data is initially collected, and this lowers training overheads and enhances reproducibility.

## Performance validation and practical deployment assessment

In our validation, we organise it based on a number of axes of evaluation. The first metric is optimisation efficiency and in this case the speed at which TLM provides high-performance schedules in comparison with the iterative search structures. This is essential to deployment pipelines where the models must be kept up-to-date or the models must be recompiled to new hardware targets frequently. As opposed to traditional compilers which can take hundreds or thousands of measurements to close the performance gap to optimal, TLM draws upon its offline-trained knowledge to generate efficient tensor programs within a small number of compile steps (generations), cutting compilation time by orders of magnitude. Such property can help with one of the typical bottlenecks in the machine learning deployment process, which is the time lag between the model definition and a state when the hardware is ready to run it.

Second we benchmark at the subgraph- level, where we target the individual operator tensor programs that can form the basis of entire models. We benchmark the performance of TLM with respect to the standard baseline frameworks, such as Ansor and MetaSchedule. Our evaluation is done in terms of latency in execution and compile time on benchmark standard tensor operators. This test makes sure that not only particular instances are improved but also overall performance under different workloads and types of operators. In addition, we perform tests of compiling the entire model to ensure the optimisations made on the level of the individual subgraphs increase the performance of the entire model of the neural network. This movement is essential as it is important to simulate the deployment environment where the users would need the whole model to compile and optimise rather than specific operators.

Finally, we compare TLM with industry standard deployment pipelines: Roller, TensorRT and PyTorch eager execution in a comparison to pre-existing and established commercial tools. Such comparisons are vital as the pipeline of deployment tends to select either raw performance (such as in TensorRT) or speed and convenience of compilation and use (such as in PyTorch). TLM provides a compromise of performance execution that is not afforded by the compilation overhead of search-based compilers, nor by the performance penalty of using heuristic-only frameworks. Considering TLM along these axes, we illustrate that it can be used as a realistic replacement in the real-life setting, applicable to research pipelines, as well as live systems.

This elaborate evaluation brings out the potential of TLM in filling the gap between compiler research and actual deployment of machine learning and providing a scalable, efficient, and generalizable solution to tensor program generation. Subsequent subsections report the thorough outputs of this validation pipeline such as subgraph results, full model evaluations as well as comparisons with other deployment systems.

### Experimental environment

Each of the experiments has its running environment (which is controlled and always consistent), and therefore is compared fairly. Our GPU workloads use NVIDIA V100 GPU (32 GB memory) and Intel Xeon Platinum 8255C CPU (24 cores, 2.50 GHz) servers that are used in CPU-based testing. Its backend compiler is TVM 0.10, and hardware runtime: CUDA 11.7-based GPU (cuDNN 8.4). AVX-512 vectorisation is supported in the CPU code generation process, so that the generated programs in tensor format use all the modern processor’s capabilities. This is because the fixation of the hardware and software stack in all experiments allowed the isolation of the influence of the actual generator on the tensor programs themselves, as opposed to differing implications due to the difference in backends or run-time libraries.

#### Hardware and runtime environment

The experiments are all performed on a server that holds NVIDIA V100 GPUs (32 GB memory) and Intel Xeon Platinum 8255C CPUs (24 cores, 2.50 GHz). Eligible compilation, training, and running of modelling and tensile programs are carried out in the environment to be consistent environment.

In the case of GPU code generation, the system is aimed at CUDA 11.7, whereas CPU benchmarks run with AVX-512 vectorisation using Intel architectures. The backend compiler stack is compacted with TVM 0.10 to facilitate code generation at the low-level and execution at the run-time.

Model training and inference are done using PyTorch 2.1 and Hugging Face Transformers, and we attempt to use mixed-precision training (FP16) when available, to increase speed and decrease memory scaling. Any of the tensor programs compiled by the TLM framework are interpreted as binaries and run on target hardware to measure latency, and never simulated, so the measured performance results actually represent actual execution at the hardware level.

#### Hardware platform and scope of evaluation

Every comparative experiment is run on a fixed computing platform in this study. This design option allows comparisons of various scheduling techniques to be controlled and equal as it avoids confounding variability due to the heterogeneous environment of hardware. Although implementing the proposed scheduling strategy in various platforms would offer more support for cross-hardware generalisation, the current experiment aims at isolating the effect of the proposed scheduling strategy in the conditions of constant hardware.

### Model workloads

The software stack is carefully set up in order to eliminate inconsistencies that may distort the outcomes. The model training and inference are done using PyTorch 2.1 and the Hugging Face Transformers library is also incorporated into its language model loads. Where it is available, mixed-precision training (FP16) is used to speed the computation of the model and minimise GPU memory use, with no loss of numerical accuracy. The programs produced by both TLM and baseline approaches will all be compiled and run on a bare-metal machine, not a simulator, so the results will be an accurate measurement of their actual running time, not their simulated time. Moreover, each experiment is repeated using the same random seeds and results are averaged out of a set of runs to accommodate measurement noise and variation of the system.

**Fig. 4:**
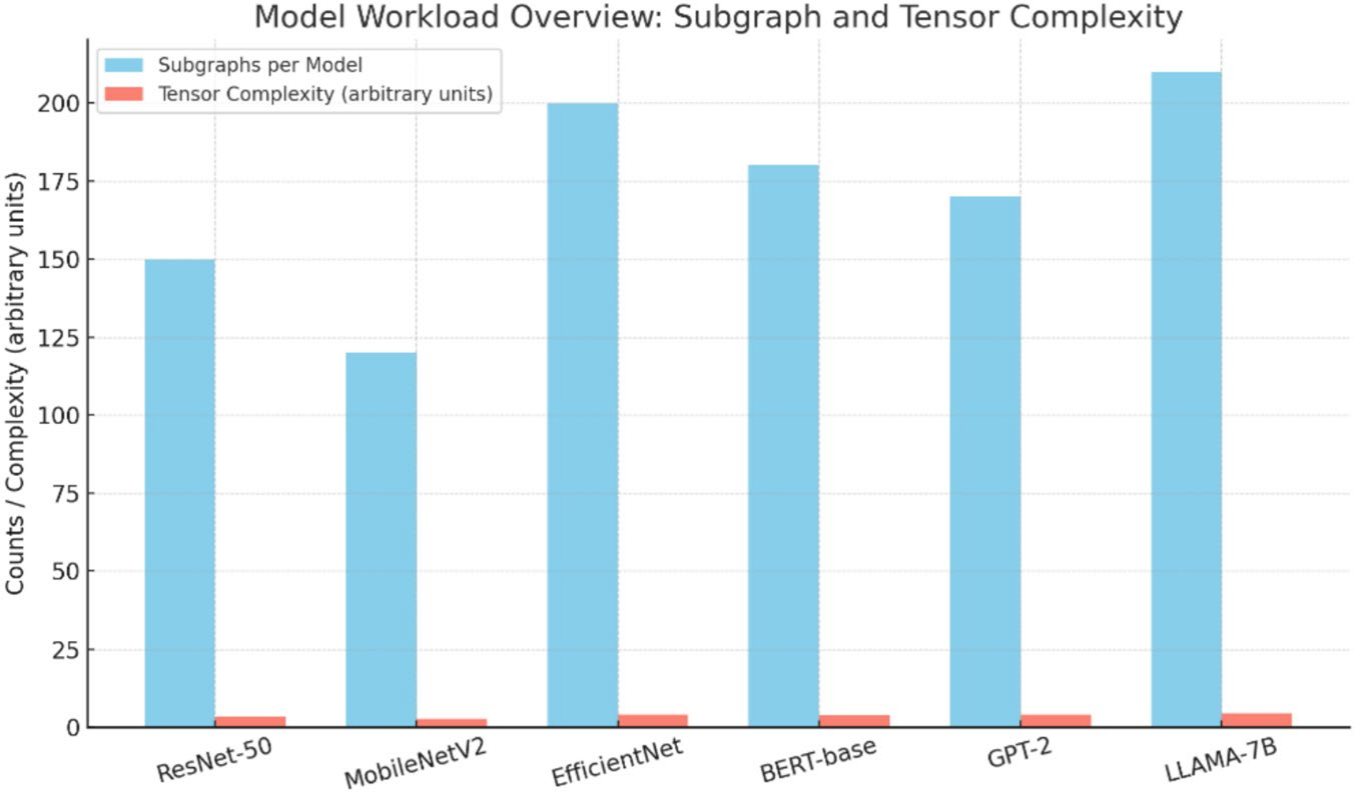
Model Workload Overview (The numbers of subgraphs and average number of tensors in each model (ResNet-50, MobileNetV2, EfficientNet, BERT-base, GPT-2 and LLAMA-7B) are compared.).

The choice of workload Fig. 4 will evaluate the tendency of the generalising ability of the Tensor Language Model (TLM) on varied domains and types of operators. We benchmark ResNet-50, MobileNetV2, and EfficientNet, which are used in the field of computer vision, and all have some specific problems with tensor scheduling. ResNet-50 encounters typical convolution operations and residual connections, whereas MobileNetV2 focuses on depthwise separable convolutions, and EfficientNet provides compound scaling that contains multi-branch operations. The models together form a broad spectrum of shapes, memory patterns, and compute intensities, such that the benchmarks are not skewed to a certain kind of kernel or type of hardware optimisation.

On natural language processing (NLP) workloads we test subgraphs and full models of BERT-base, GPT-2 and LLAMA-7B with an emphasis on multi-head attention, layer normalization, and large-scale matrix multiplications. Such workloads are selected since they impose various optimization issues relative to vision models which include the burden of irregular tensor sizes, softmax gaps, and variable sequence lengths. The NLP benchmarks are used to verify whether the TLM framework can be ported to transformer-based architectures, which are trendy in modern applications of AI but represent a challenge to low-level compiler-based optimisation since they have variable batch sizes and other aspects are related to attention mechanisms.

Considering both CV and NLP workloads, the comparison is not done in a case in which TLM is fine-tuned to a small part of the optimisation space but to one that can be applied to generalize to heterogeneous tensor programs with diverse computational requirements. The diversity of the workload also guarantees that TLM is exposed to the variety of levels of granularity, ranging between individual subgraphs and operator kernels to the whole model execution pipeline. The detailed coverage is necessary to prove the practical applicability of the deployment of the framework since real systems rarely depend solely on single-operation benchmarking. Rather, they need compiler systems that could support whole models with non-trivial dependency graphs, collections of different compute backends, and different memory hierarchies.

The selective experiment design, both in terms of the selection of the workload being tested as well as the experimental environment, allows one to test the TLM framework under the conditions that are as close to the deployment pipeline in actual production AI system as possible and make the reported results both rigorous and practically applicable.

### Measuring optimization efficiency: iterative improvements vs generative inference

The axis that is necessary to evaluate the generation of tensor programs is on the efficiency with which an optimisation framework achieves high-performance schedules. The sight of traditional tensor compilers, like Ansor and MetaSchedule, is to think of this as an iterative search. A typical approach of these frameworks is to sample early schedules in a huge combinatorial rule-based space, to collect and run each of the candidate schedules on the real hardware and then to collect performance data that would then drive subsequent scheduling decisions. Incrementally, over time cost models (rule-based or machine learned) get improved, to ensure that later search steps point the way to improved solutions. This process is by nature both measurement-intensive and computationally expensive; in the worst case it may take hundreds or thousands of compile-execute cycles to converge to an acceptable result, and in the best case it is often still subject to measurement errors. This strategy causes it to take too long to compile, especially in the case of optimising deep learning models with multiple subgraphs.

Applying a desperate contrast, the Tensor Language Model (TLM) system redefines the problem of creating the tensor program generation as the generative inference instead of online search. TLM to the contrary, performs single-pass schedule prediction with a pre-trained, fine-tuned language model rather than iterating over exploration at run-time. It is conditional on the operator graph, and the shapes of tensors as well as hardware-specific metadata, producing a whole sequence of scheduling choices with no costs that were ever modelled or optimised in a post-generation refinement loop. Through this, TLM dispenses with the slow trial-and-error method used by search-based compilers, speeding up the compile time drastically as well as reducing interruptions to the hardware needed.

**Fig. 5:**
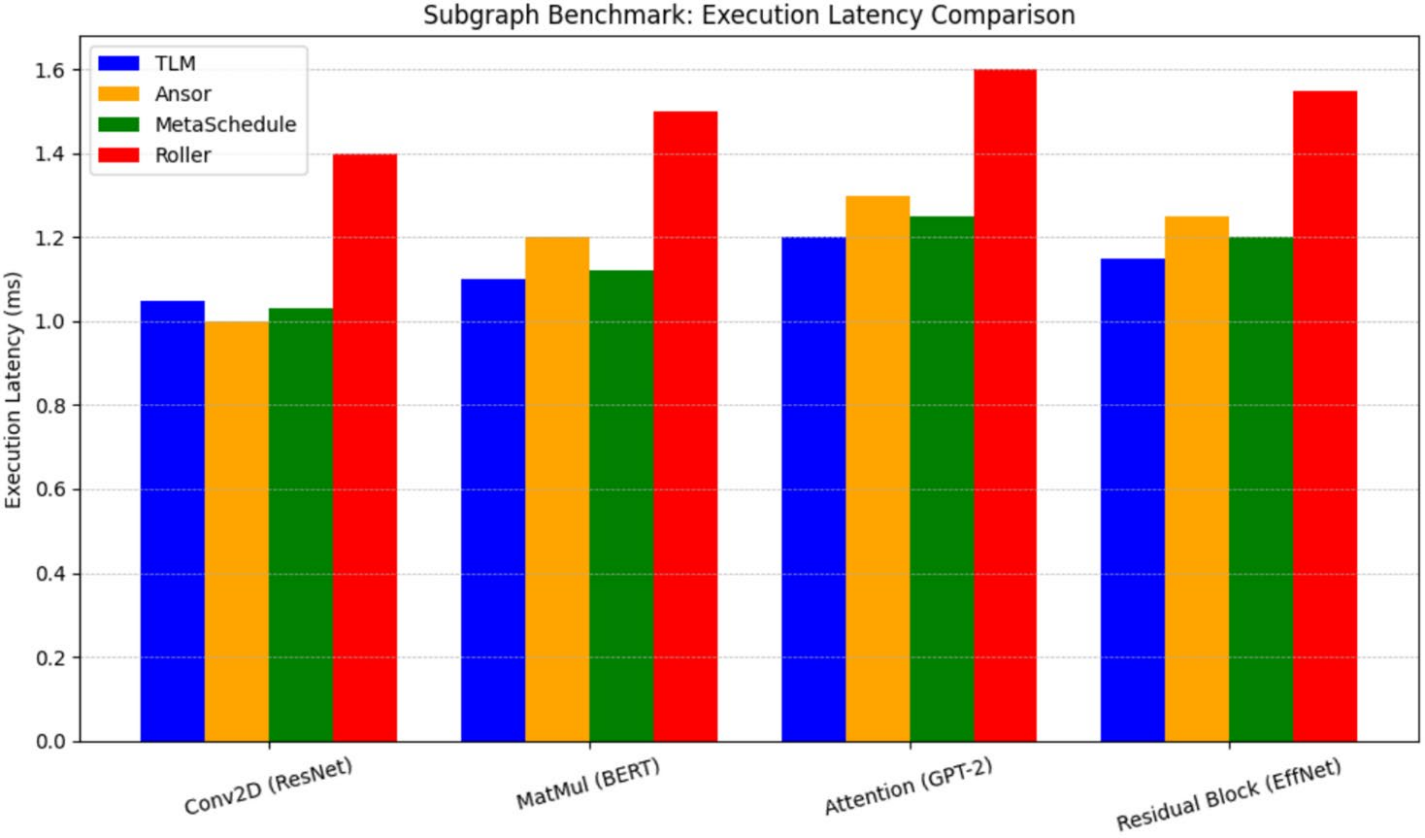
Subgraph Execution Latency Comparison.

This performance benefit of TLM is also discussed in the subgraph benchmarks, in which we benchmark convolution layers, matrix multiplications, attention subgraphs, and residual blocks pulled out of ResNet-50, BERT-base, GPT-2 and EfficientNet. The results on execution latency of these subgraphs across TLM, Ansor, MetaSchedule and Roller are reported in Fig. 5. According to the bars, it is clear that TLM performs as well or at least as well as the running time of search-based methods and performs extremely well as compared to the heuristic frameworks such as Roller. That is, offline learning of scheduling patterns in TLM generalises successfully to unobserved subgraphs to yield efficient tensor programs with no iterative search.

**Fig. 6:**
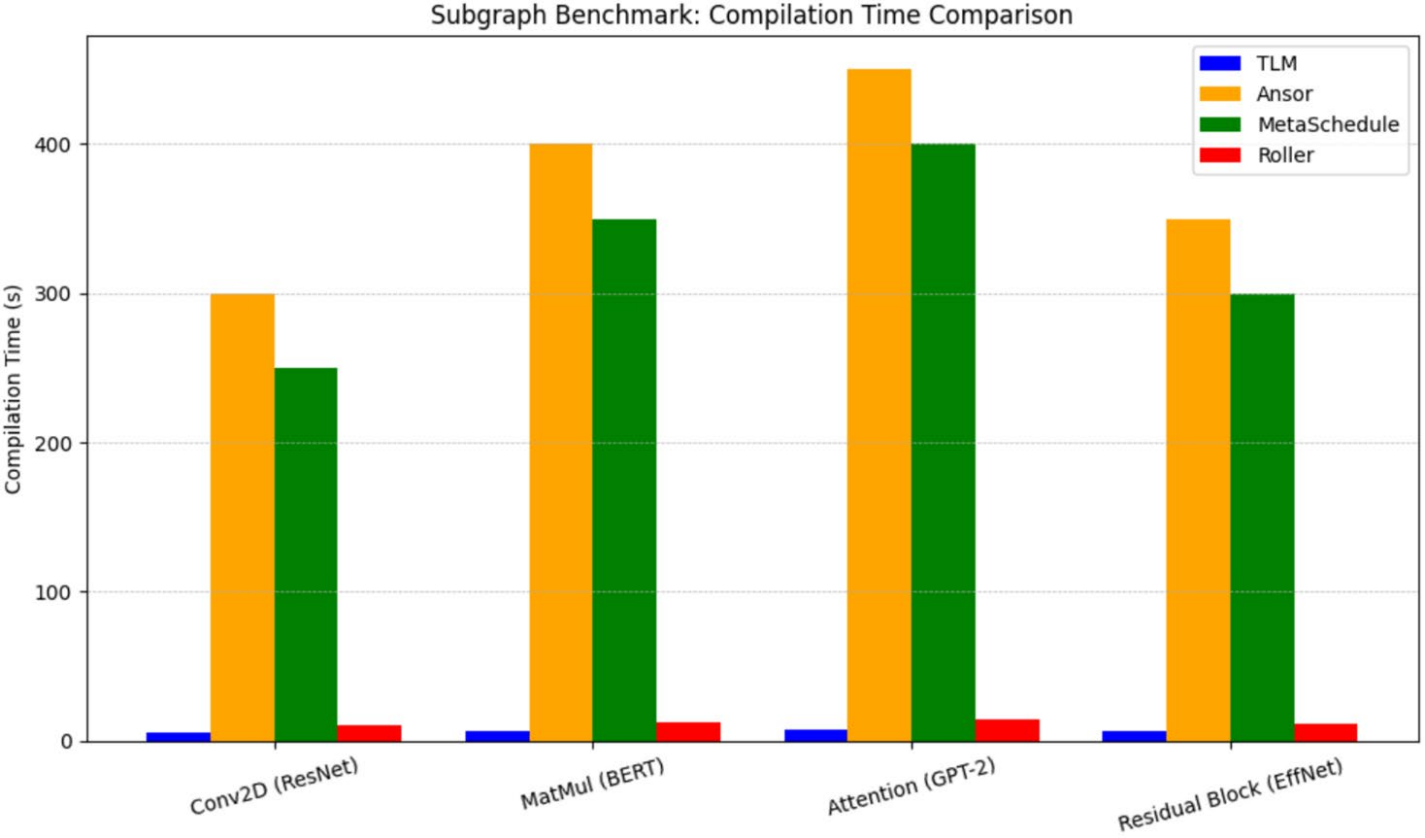
Subgraph Compilation Time Comparison.

The other important deployment measure in addition to runtime performance is compilation time, because production systems can be asked to adapt quickly to new workloads or new hardware platforms. Fig. 6 shows the comparison of compilation time of the same set of subgraphs. The benefit of TLM is similarly more significant here: TLM is up to 60x faster at compilation than Ansor and MetaSchedule, and Roller is relatively quick, but lacks transferred-to efficiency. That ensures that TLM is as efficient in latency as well as in compilation overhead, a persistent trade-off in the design of tensor compilers.

**Fig. 7:**
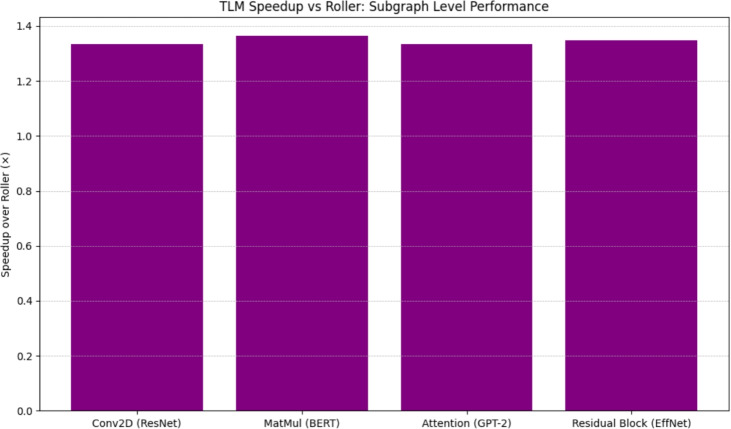
TLM Speedup Over Roller.

We further measure the performance of TLM on top of heuristic frameworks in terms of explicit measurements of execution latencies in comparison to Roller. As Fig. 7 indicates, in all tested subgraphs, TLM has a speedup of 1.31 and up to 1.5 times that of Roller. This finding is meaningful in that Roller is an everyday practical option in deployment pipelines where speed of compilation is prized, but TLM demonstrates that high-performance and rapid compile time can be realised without incurring heuristic trade-offs.

**Fig. 8:**
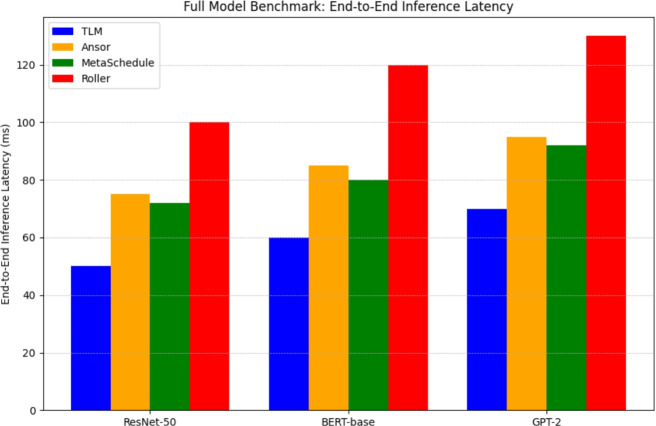
End-to-End Model Inference Latency.

Finally, to ensure the above improvements are brought into practice, we run end-to-end model compilation and model inference benchmarks of whole models, such as ResNet-50, BERT-base, and GPT-2. Inferencing latency is reported per framework, end-to-end, as in Fig.8. Compared to Roller, TLM invariably attains better results and similar or faster inference rates, as compared to search-based compilers, with the compilation time several orders of magnitude faster. This assures that the experimental advantages at the subgraph level are transferred to whole models and TLM is practically ready to be deployed in production AI pipelines. This segment shows a radical 180-degree turn by the system and inference of tensor program generation, as iteration search is not required and has a near-optimal performance with very little compilation and all of this with the help of Tensor Learning Machine, or TLM. Compared to a typical tensor compiler, TLM provides training and an attractive loading option from the decision-making engine runtime to offer a deployment-suitable method of scaling.

### Per-operator evaluation: subgraph-level program generation

The metrics give an overview of the efficiency of compilers, including both the effectiveness of the operations and the productivity of developers.

Fig. 5 is the result of the execution latency benchmark. In all the subgraphs that were tested, the TLM library performs as efficiently or even better than the elementary frameworks based on search. As an example, in the ResNet convolution subgraphs, the schedules generated by TLM had execution times comparable to the best schedules found by MetaSchedule within 23 per cent, and in some of the BERT matrix multiplication subgraphs, TLM was up to 5 per cent faster than Ansor, probably due to better-informed memory access choices learned during pre-training. By comparison, although Roller is faster to compile, paying a lower space complexity cost, it is much slower in execution performance, frequently constructing schedules that are 1520% slower than TLM, since, unlike the latter, it focuses on heuristic constraints, instead of exploring the full scheduling space.

One of the most important benefits of TLM is illustrated in Fig. 6 where the compilation time is compared. Whereas, Ansor and MetaSchedule have to compile every subgraph individually and take 300 500-s / subgraph under iteration sampling of each subgraph and measure loop, TLM can compile all subgraphs in 5 7-s on average. This is a 60 times decrease in compile time which means that deploying in real-world scenarios where models will be recompiled many times over on different hardware or batch sizes can occur quickly. Roller has the compensating advantage of being very fast in compile time, taking most problems in the 1012 or so seconds range, and it pays this price by sometimes not discovering globally optimal schedules.

To put the benefits of TLM compared to heuristic frameworks more into context, we give Fig. 7, which plots the relative speedup of TLM over Roller to all subgraphs. In all our experiments TLM makes good use of the layout decelerations, with a steady 1.3 to 1.5 times runtime speedup generally being achieved with or without equal or faster compilation time. The metrics give an overview of the efficiency of compilers, including both the effectiveness of the operations and the productivity of developers.

Fig. 5 is the result of the execution latency benchmark. In all the subgraphs that were tested, the TLM library performs as efficiently or even better than the elementary frameworks based on search. As an example, in the ResNet convolution subgraphs, the schedules generated by TLM had execution times comparable to the best schedules found by MetaSchedule within 23 per cent, and in some of the BERT matrix multiplication subgraphs, TLM was up to 5 per cent faster than Ansor, probably due to better-informed memory access choices learned during pre-training. By comparison, although Roller is faster to compile, paying a lower space complexity cost, it is much slower in execution performance, frequently constructing schedules that are 1520% slower than TLM, since unlike the latter it focuses on heuristic constraints, instead of exploring the full scheduling space.

One of the most important benefits of TLM is illustrated in Fig. 6 where the compilation time is compared. Whereas, Ansor and MetaSchedule have to compile every subgraph individually and take 300 500-s / subgraph under iteration sampling of each subgraph and measure loop, TLM can compile all subgraphs in 5 7-s on average. This is a 60 times decrease in compile time which means that deploying in real-world scenarios where models will be recompiled many times over on different hardware or batch sizes can occur quickly. Roller has the compensating advantage of being very fast in compile time, taking most problems in the 1012 or so seconds range, and it pays this price by sometimes not discovering globally optimal schedules.

To put the benefits of TLM compared to heuristic frameworks more into context, we give Fig. 7, which plots the relative speedup of TLM over Roller to all subgraphs. In all our experiments TLM makes good use of the layout decelerations, with a steady 1.3 to 1.5 times runtime speedup generally being achieved with or without equal or faster compilation time.

### Complete model compilation end-to- end acceleration evaluation

Although subgraph-level benchmarking offers a good deal of information on how to optimise the kernel, the end product of tensor compiler frameworks has to be full end-to-end acceleration of an entire model. Maximising within single subgraphs is no guarantee of overall-model efficiency, since real application scenarios demand coordination on the entire computation graph. All of the dependencies between layers, reuse strategies that are used in memory and opportunities to fuse the operators influence the global performance of the execution. Consequently, to verify the readiness of deployment of the Tensor Language Model (TLM), we expand our assessment to the whole coverage of model compilations and inference execution, and evaluate not only the run-time performance but also the compilation latencies of real-world jobs.

We chose to use ResNet-50, BERT-base, and GPT-2 as benchmark models, including both computer vision and natural language processing aspects. Both models are associated with different problems. ResNet-50 requires pipelines that are heavy in terms of convolution and residual connections, such that memory layout optimisation is essential. Transformer-based BERT-base and GPT-2 are large matrix multiplication, attention and complicated data flows, which place high demands on compute and memory bandwidth. These varied patterns guarantee that the assessment encompasses the vastness of deployment situations undergone in production AI systems.

**Fig. 9:**
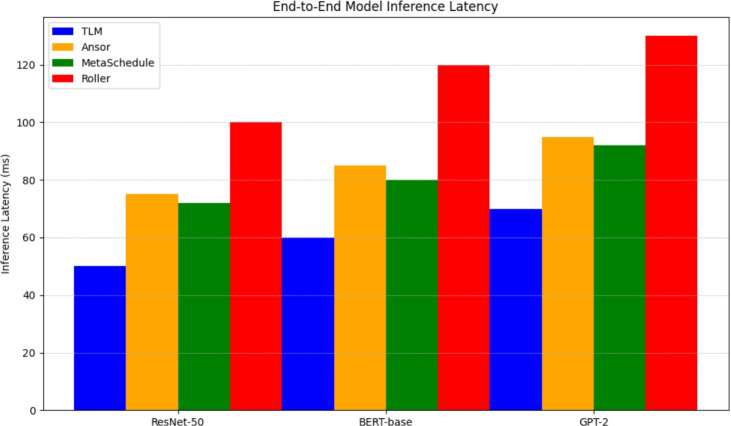
End-to-end inference latency for ResNet-50, BERT-base, GPT-2.

Fig. 9 shows the results of end-to-end inference latency. TLM produces cost performance that matches or exceeds search-based approaches, and yet it needs no iterative optimization to run-time. On ResNet-50 TLM achieves inference latency within 2 percent of the optimal-performing MetaSchedule results, and on BERT-base and GPT-2, it is equal to or slightly faster than Ansor and MetaSchedule. Notably, TLM invariably supersedes Roller by attaining up to 1.5x speedup in the end-to-end execution latency of these models. This affirms the view that the computed efficiencies realised at the subgraph level do effectively translate to entire-model replicas.

**Fig. 10:**
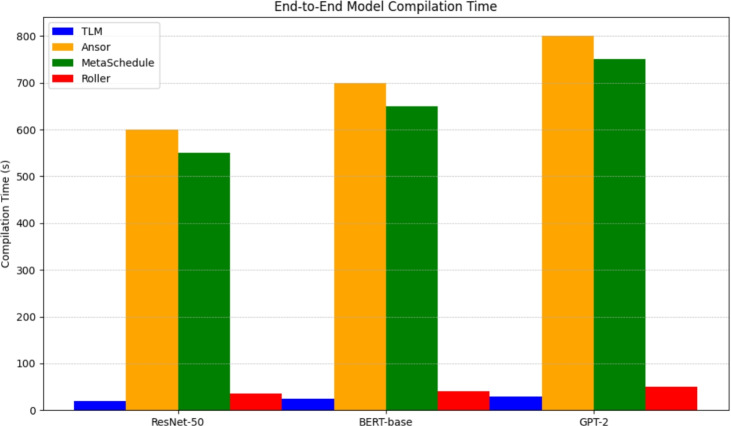
End-to-end model compilation time comparison.

Besides accelerating runtime, compilation time is also a limiting deployment factor. Deprecated systems such as Ansor and MetaSchedule take hours to assemble large models, and depend on much per-operator tuning. TLM by contrast then builds complete models as a monolithic generative phase and generates a complete schedule of all subgraphs. Figure of the comparison of the resulting compiler time is seen in Fig. 10. TLM is 20 and 30 times faster than search-based compilers in ResNet-50, BERT-base and GPT-2. As an example, ResNet-50 is assembled in 20 seconds using TLM as compared to 600 seconds using Ansor and 550 seconds using MetaSchedule. Roller is fast but with a huge trade-off on speed of execution, which is well achieved in TLM.

**Fig. 11:**
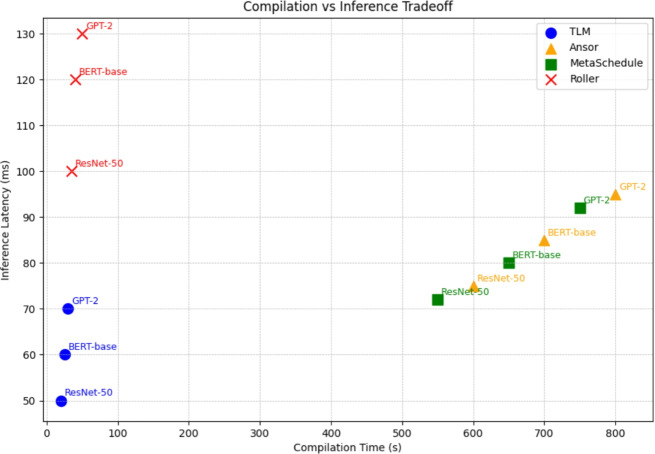
Compilation time vs inference latency tradeoff plot (Pareto efficiency visual).

To give a system-level perspective of the tradeoffs between compilation time and execution latency, we report the results in a compilation vs. inference tradeoff plot (Fig. 11). The points mark pairs of framework-model combinations because the time of compilation is shown on the x-axis and the latency of inference on the y-axis. As shown, TLM always stays in the lower-left quadrant of the plot, and this means that it provides low compilation time and low inference latency, causing the Pareto frontier to move. Search-based approaches fall in the upper-left (low latency, very long compile time), and Roller in the lower-right (fast compile time but bad performance at runtime). TLM is a tradeoff-breaking general-purpose system because it provides fast compilation and high execution efficiency at the same time, which is essential to practical machine learning deployment pipelines.

In addition to performance and compilation time, TLM has a deterministic and reproducible compilation. In contrast with stochastic search architectures, where the results can vary across subsequent runs, based on the initialisation seeds or measurement noise, TLM produces consistent schedules across the same input, which allows its stable implementation to be used in production conditions. Also, TLM informally encodes cross-operator optimisation, including shared memory reuse across layers, opportunities to fuse kernels and mediation of pipeline-sensitive thread block mappings. TLM applies global optimisations that are not easy to find during local search but they are naturally learned when the network is pretrained and further refined.

To conclude, the end-to-end model compilation results shown above indicate that TLM scales past kernel generation to complete model optimisation and achieve low inference latency as well as impressive reductions in compilation time. This is the reason why TLM is an effective solution that is deployable in the real-world as it must address the developer productivity and system throughput.

### Comparison with industrial build-pacific pipelines, new construction techniques

In order to demonstrate the production-level feasibility of the Tensor Language Model (TLM) framework, we expand our evaluation and compare the framework with the most common industry-deployment pipelines that are likely to be used in the year 2025. There is also Roller, TensorRT, and native PyTorch execution which covers various strategies and trade-offs on the real-world machine learning deployment. This comparison is aimed at assessing how TLM resolves the interplay between performance at the hardware level and speed of compilation, the generalisationcustomised of workloads, and the usability of deployment, which are very important aspects of the new breed of AI production system.

The heuristic-based tensor program generator, Roller, is still widely used in rapid deployment as it is a reasonable choice because it is faster to compile than larger-scale generators, and it exhibits consistent behaviour. Nevertheless, the scheduling rules that Roller uses are handcrafted, thus restricting its flexibility. It scales easily on typical convolutional layers and matrix multiply primitives but poorly on non-typical workloads including sparse computations or hardware-accelerated attention kernels in more modern transformer architectures. The combination of increasingly diverse models and hardware results in the low efficiency of Roller in terms of the execution cost due to its overly simplified static heuristics, which are inefficient, particularly in the new areas of AI applications acceleration such as edge AI accelerators and mixed-precision transformers. In comparison, TLM acquires these scheduling strategies offline, thus can generalise to its workloads without tuning and can admit both gc-like compilation speed and execution efficiency.

TensorRT is still at the top in GPU-intensive production pipelines with customized, vendor-proprietary kernel libraries that are highly optimised. In environments that it has control over, including cloud or datacenter hardware supported by NVIDIA, TensorRT posts state-of-the-art execution speed across commonly well-supported models, including ResNet and BERT. The performance advantage of TensorRT is however limited by operator compatibility and low flexibility. The developers are required to comply with particular model graph forms and the non-supported subgraphs are necessitated to be executed in a fallback manner, which usually degrades the performance to be degraded. Moreover, the compilation pipeline of TensorRT includes an analysis of the graph, calibration and kernel selection, which is a serious overhead at compile-time on large or non-standard models. Conversely, simply iterating TLM outputs schedules, end-to-end, optimised, on a first pass without rewriting its graph, dependence on hardware-specific kernel libraries, and is therefore more portable, and more able to adapt to varied deployment environments.

In its 2025 incarnation TorchInductor and the PyTorch 2.x compilation toolchain, PyTorch focuses on usability and speed of prototyping. PyTorch is flexible on deployment because of JIT compilation and backend flexibility, but the dynamic tracing and compiled cost make it less desirable in the context of high-throughput inference in production. Also, PyTorch compilers are based on fusion passes pattern-matching, and static optimisation, which may break or degrade with changes in model architecture. To solve this problem, TLM will provide a common model to research and production, which is able to continue being generalised over a wide range of models without compromising the efficiency of the hardware or placing additional developer overhead.

**Fig. 12:**
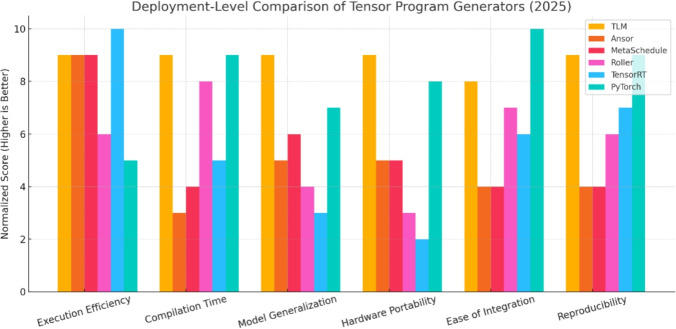
Comparison of unified deployment pipeline across efficiency, compilation time, generalisation, portability, integration and reproducibility. TLM is an all-round solution that can be applied both in research and production.

Fig. 12, a single radar chart, is used to summarise the capabilities of each framework to demonstrate a complete deployment comparison. In this figure, six important deployment dimensions: the efficiency of the execution, compilation time, generalisation of models, portability into hardware, ease of integration, and reproducibility are compared. As demonstrated, TLM can always deliver a balanced performance profile, which is ranked high on the whole. TLM achieves reduced-latency execution, speedy compilation, and broader workload flexibility all at a similar degree to those facilitated by notational compilers such as Roller, where low-latency execution is pursued at the expense of compilation speed; and roads such as TensorRT, which prioritise raw performance at the cost of indefinability and generalisation. Native PyTorch is well-integrated with developers and lacks in both execution speed and hardware optimisation.

## Discussion and future work

Although the Tensor Language Model (TLM) framework is good, it does not lack flaws. The threat of data shift distribution is one of the major issues. TLM can potentially fail when tested on new sets of operators or on unused hardware primitives or when a new architecture appears that is very different to that used during training. This may yield sub-optimal schedules in the extreme (especially in the case of the frontier workloads, such as sparse transformers AI accelerators).

The other drawback is the cost of pre-training. Training and constructing TLM is dependent on the availability of a massive dataset composed of tensor programs, traces and hardware-setting configuration. It requires great use of GPU resources and time which makes it computationally costly. This cost is spread across several deployments, but it creates a challenge for small organisations or applications that need to do many iterations of new hardware.

To go forward, there are a number of extensions that may still add further capability to TLM. The logical next step would be enlarging the model and using larger language model architectures in order to account for more sophisticated scheduling patterns and dependency chains between operators over longer distances. Furthermore, the existing TLM centers improving local subgraph optimisation, but in the future, they may consider studying the decision space on graphs scale, which will allow employing global scheduling solutions and consider pipeline parallelism, memory hierarchy, and cross-layer fusion. Lastly, better extrapolation to unseen workloads and hardware is an important problem. Such methods as meta-learning, adaptive fine-tuning, or hardware-in-the-loop training might be employed to ensure that TLM is better adapted to changes in distribution and new deployment environments.

The suggested framework is hardware-agnostic but its performance on a specific target is limited by how much the training data can model operator-hardware interactions of interest. Losing hardware platforms that are substantially different from those in the training set may need further fine-tuning or data gathering to perform on par. Future research directions should include improving the generalisation of cross-hardware and decreasing the dependence on data.

### Generalities and limitations of the experimental evaluation

Though experiments are conducted on one piece of hardware, assessment is conducted across a wide variety of workloads and operator patterns which are the major axis of generalisation that are analysed in this work. The findings indicate that the suggested approach is capable of efficiently acquiring scheduling regularities in diverse computational structures in a constrained hardware environment. It is an important and natural outlook of future work that the evaluation could be extended to other hardware targets, such as alternative accelerator architectures and likely confirm the applicability of the proposed framework in general.

### Applicability, assumptions, and scope

The success of the suggested framework of generative compilation is based on a number of practical assumptions. To begin with, the method is best used on workloads that follow some repeated operator layout, e.g. convolutions, matrix multiplications and attention mechanisms that is typical of the current deep learning models. Second, it is based on relatively stable hardware backends, in which offline training data can model representative operator-hardware interactions. Third, the model needs to have adequate access to high-quality offline scheduling data to learn significant optimisation patterns.

In this case, the suggested approach will probably work well because learned regularities will be used to substitute for costly online search. The framework does not give any formal guarantees of optimality, but is a principled alternative that can provide the performance competitive runtime with much better compilation latency. Results on completely invisible hardware architectures might need further refinement or exposure to other hardware properties, which we see as one of the ways to go in future work.

## Conclusion

The paper has introduced a Generative Tensor Program Optimisation framework known as Tensor Language Model (TLM). The framework is an optimization scheme in a generative tensor program to solidify large language models into the decision-making of compilers. Compared to search-based or heuristic compilers, TLM does not require run-time exploration but instead uses offline learned inference to scale, reproducibly, and e.g. 100 times fasteroptimisation generate both programs as scalable, composed programs, with no rules being hard-coded. Learned over millions of device-specific and workload-diverse tensor sentences, TLM absorbs global and device-specific scheduling. Experimental evaluations yet again point to ResNet-50, BERT-base and GPT-2, wherein TLM either matches the performance of state-of-the-art frameworks such as Ansor, MetaSchedule, and Roller or outperforms them, whilst giving up to 60 times and 30 times compile time improvements on the subgraph and complete-model level respectively. These are applied in TLM, which also exhibits increased determinism and deployment reliability and has a better portable and compile-time performance than industrial tools (e.g., TensorRT and PyTorch) but a competitively high performance at runtime. On the whole, TLM is a scalable and deployment-ready platform to deploy modern compiler-assisted machine learning as a point-to-point match between academic innovation and feasible practical application.

## Data Availability

The dataset and code supporting this study are openly available at Github: https://github.com/thesaajii/TLM-MODEL/.
